# #BetterHealth: A qualitative analysis of reactions to the UK government’s better health campaign

**DOI:** 10.1177/1359105320985576

**Published:** 2021-01-10

**Authors:** Catherine V Talbot, Dawn Branley-Bell

**Affiliations:** 1 Northumbria University, UK; 2 Bournemouth University, UK

**Keywords:** communication, obesity, public health, weight

## Abstract

This study examined reactions to the UK government’s Better Health campaign through a thematic analysis of tweets. Four themes were generated: Embracing Better Health; There is no Better Health without mental health; Inconsistent messaging; Only a surface-level solution. Findings suggest the campaign is problematic, given its lack of consideration for mental health and wider societal factors that contribute to obesity. The campaign could exacerbate mental health difficulties for individuals with eating disorders due to its focus on weight and perceived fat-shaming approach. Recommendations are made to develop future campaigns that avoid negative public responses, minimise harms, and maximise intended benefits.

## Introduction

The ‘Better Health’ campaign was launched by the UK government on 27th July 2020 (Public Health England, 2020). This national campaign encouraged individuals to make healthier lifestyle choices, by losing weight, getting active and quitting smoking. A range of tools were available to help people manage their health, including mobile applications (e.g. NHS Smokefree; Couch to 5K), online support groups, virtual fitness videos, a body mass index (BMI) calculator, and offers with organisations who provide weight loss plans (e.g. Slimming World). The campaign launched during the COVID-19 pandemic and was driven by data showing that obese people are significantly more likely to become seriously ill and be admitted to intensive care with coronavirus compared with individuals with healthy BMIs (Docherty et al., 2020; Hussain et al., 2020). Consistent with this, the general population have reported facing difficulties staying healthy during the pandemic, with Duffy (2020) finding that almost half of respondents reported putting on weight and 29% reported increased alcohol consumption.

Responses to the Better Health campaign have been mixed, with Sport England (2020) and the British Heart Foundation (2020) welcoming measures that aim to tackle obesity, such as the 9pm watershed on fast food adverts. In contrast, the campaign has also received some negative press coverage (Mead, 2020; Wilson, 2020) and reactions from eating disorder organisations (e.g. BEAT, 2020) who argue that the campaign may be damaging for some people. This is substantiated by research showing that people with eating disorders have been negatively impacted by ‘a public preoccupation with food, weight gain and exercise’ during the COVID-19 pandemic (Branley-Bell & Talbot, 2020, p. 10). Insight into public reactions can identify whether the campaign is perceived as socially excluding or damaging to some populations. It is important to know how the campaign is being received by the public more widely. These insights can help to guide the design of future campaigns to maximise positive behaviour change, and minimise the potential for unintended negative impacts.

Social media provides a valuable source of information about public attitudes, opinions and even behaviours (Snelson, 2016). Twitter is one of the widely used social media platforms and is often regarded as a platform used to publicly voice opinions (Branley & Covey, 2017; Highfield, 2016). Consistent with this, researchers have used tweets to evaluate health campaigns (e.g. Schlichthorst et al., 2018), yet to our knowledge, this method has not been used to evaluate the Better Health campaign. Our study addresses this gap and uses public tweets to examine reactions to the Better Health campaign.

## Methods

### Data collection and sampling

Ncapture was used to collect tweets posted between 27th July 2020 and 7th August 2020 containing at least one of the following search terms: ‘#BetterHealth’; ‘Better Health campaign’. Tweets were collected across this 10-day time period to capture immediate reactions to the campaign and also to ensure the sample was not unweildy. Ncapture identified 4606 tweets in total, including 3266 retweets.

As we were primarily interested in the content of individual tweets, retweets were excluded from the sample. Only tweets written in English were included in the sample. We also excluded tweets from health organisations advertising the campaign (e.g. Public Health England, the National Health Service and National Health Trusts) as these do not represent public reaction. Company advertisements were also excluded from the sample. The final sample included in the qualitative analysis comprised 181 tweets (approximately 13.5% of individual tweets collected by Ncapture).

### Analysis

Tweets were imported into NVivo12 and analysed thematically, following the six steps outlined by Braun and Clarke (2006): data familiarisation; generating initial codes; searching for themes; reviewing themes; defining and naming themes; producing the report. The first author began by familiarising herself with tweets and coded the entire dataset, identifying initial themes. These themes were then shared with the second author who provided critical feedback. Changes were made to themes where appropriate and the analysis was refined until both authors agreed upon the finalised themes.

### Ethics

Ethical approval was obtained from the Northumbria University ethics board [26267]. The British Psychological Society code of Ethics and Conduct (2014) states that unless consent has been sought, observation of public behaviour must take place in spaces where individuals would expect to be observed by strangers. Tweets can therefore be viewed as public behaviour. However, to promote the anonymity of users it is good practice to paraphrase quotations of tweets to reduce the likelihood of users being identified using search engines. Therefore, all quotes in this paper have been paraphrased in a manner that retains the original meaning, with both authors agreeing on the phrasing of each tweet.

### Data availability

Data are not available for this study given that account holders could be identifiable if it were made accessible.

## Results

Our thematic analysis generated four themes: Embracing Better Health; There is no Better Health without mental health; Inconsistent messaging; Only a surface-level solution. Despite the campaign focusing on different factors associated with health, tweets tended to focus on weight loss and this is reflected in our themes.

### Embracing better health

Researchers have found that social media can provide individuals with access to supportive communities that can help with weight loss (May et al., 2016). This was also evident in our sample, with users documenting their weight loss journeys and sharing personal experiences on Twitter. For example, users often described the positive physical and mental changes that accompanied weight loss:

*After my heart attack, I could have stayed as I was, but I didn’t. I changed my diet and walked daily, it wasn’t difficult, I didn’t do anything special but as a result, I lost five stone in a year, which helps my heart, relieves some pressure, small changes #BetterHealth*

Some users felt that the campaign engendered a sense of community among the population, with one person tweeting:

*Yesterday, I started a vegan diet. I feel like I’ve joined a slimming club with the whole country. I like it. #BetterHealth*

Whilst others tweeted that they felt motivated by the campaign:

*I’m very encouraged by #Betterhealth. The NHS ‘Better Health’ campaign encouraging weight loss is a good idea, it’s commendable.*

This suggests that the campaign was a positive influence for some users, motivating them to adopt healthier lifestyles.

### There is no better health without mental health

Unfortunately, other users tweeted that there was a lack of consideration for mental health by the campaign, with one person advocating: ‘*there is no #BetterHealth without mental health*’. This is particularly problematic, given that the campaign was established during the COVID-19 pandemic – a time when mental health problems were more pronounced (Pierce et al., 2020):

*It isn’t #BetterHealth though? It’s #WorseMentalHealth. Mental health is important and I wish the government would not ignore it.*

Above and beyond the campaign simply overlooking mental health, many felt that it could actually be *detrimental* for mental health, particularly for those with eating disorders. Users commented that the campaign was triggering of disordered eating behaviours because of its fat-shaming approach, and focus on lower weight and calorie intake as equating to health:

*#BetterHealth does not (and should not) equal being thin. Calorie counting to control your diet is known to be a big trigger if you suffer from disordered eating. Restricting food intake is not always the healthier choice.*

Many users tweeted about representations of obesity in the campaign, with some stating that the campaign was ‘*fatphobic*’, ‘*disempowering*’ and reinforcing of stereotypes:

*The NHS #BetterHealth advert is such a one-dimensional portrayal of overweight people – it depicts them as just fatties who fail when it comes to exercise!*

Fat-shaming media messages can be damaging to people with eating disorders (Spettigue et al., 2004). For example, these type of messages can enhance implicit anti-fat attitudes, which have been associated with reduced wellbeing (Ravary et al., 2019; Webb et al., 2016). Consistent with this, users commented that the pandemic posed heightened risks for people living with, or susceptible to, eating disorders. For example, one person highlighted that the general population may not notice calories on menus, but this could have detrimental impacts on individuals with eating disorders:

*I would love to know how many people, who do not have disordered thinking about food, will actually read calories on menus. Most people will just be like ‘whatever’ but those of us with eating disorders will be left feeling like we can’t eat anything.*

It was evident from the tweets that many users had lived experience of eating disorders, with some stating that the campaign would make their daily experiences more difficult:

*I am lost for words about the #BetterHealth campaign. I’m terrified for those of us who have worked so damn hard to develop a happier relationship with food. This campaign will only make this everyday battle a lot harder.*

This suggests that the campaign may pose heightened challenges for people with eating disorders – a population that has already faced significant difficulties during the COVID-19 pandemic (Branley-Bell & Talbot, 2020).

### Inconsistent messaging

In their tweets, some users perceived the UK government to be delivering inconsistent messaging. For example, one month after the campaign was established (August 2020), the government launched the ‘Eat Out to Help Out’ scheme which encouraged the public to dine at restaurants by providing discounts; helping to support the economy during the pandemic. This was perceived by many as contradictory and hypocritical, particularly as some perceived the restaurants as focusing on fast-food or unhealthy options:

*#BetterHealth campaign = because you are fat, you are causing coronavirus deaths to rise. Lose weight!! Eat out to Help out scheme = here’s 50% off your bill, take your family to KFC or McDonald’s!*

Users also pointed out unfortunate TV advert scheduling, where Better Health commercials were immediately followed by fast food or takeout adverts:

*Watching the TV, I saw the #BetterHealth campaign for the first time. The advert was im-mediately followed by an advert for McDonald’s.*

These inconsistent messages were clearly confusing for users and could potentially impact the effectiveness of the campaign. Instead, a cohesive, whole systems approach may be more effective in promoting Better Health.

### Only a surface-level solution

Obesity is a complex condition and researchers have identified genetic, lifestyle, educational and environmental risk factors (Fruh, 2017). Some users felt that the campaign fails to account for the complexity of obesity by not considering these wider societal factors:

*Already, after less than a week, I’m tired of seeing the Better Health adverts. Instead of addressing the reasons why some people find it hard to eat well, it’s just a surface level solution.*

Some drew attention to health inequalities that contribute to obesity, for example highlighting the cost involved in eating healthy:

*#BetterHealth Make fruit and veg cheaper for everyone as I find it so expensive to eat healthy! In comparison, crisps and chocolate are cheap, cheap, cheap!*

Instead of promoting calorie-counting, many users advocated for better education around health behaviours and cooking skills, and increased access to exercise facilities:

*We need to recognise the psychological reasons behind conditions, give people the skills to prepare healthy food, and access to exercise. Prescribing cycling and cutting calories misses the point! #BetterHealth is not enough.*

These findings highlight that some users felt the campaign did not account for the complexity of obesity and excluded certain groups of people, such as those who cannot afford to engage in Better Health behaviours.

## Discussion

Our findings suggest that public reactions to the Better Health campaign were mixed, reflecting offline debates about its appropriateness (e.g. BEAT, 2020). The campaign was received positively by some individuals who tweeted about the positive impact it was having on their lives, increasing exercise and motivation. Interestingly, users tweeted that the Better Health campaign engendered a sense of community among the general population. Given a recent focus on social prescribing (Drinkwater et al., 2019), our findings suggest that communities may be a valuable resource for promoting healthy lifestyles. This supports public health campaigns as a potential driver of positive behaviour change, and although weaknesses were also identified in the Better Health campaign – there are lessons to be learned. These insights can help guide the design of future campaigns to maximise the benefits and protect against unintended harms.

Despite the campaign being beneficial for some individuals, our findings show that the Better Health campaign was perceived by some to be inappropriate, shaming, dismissive of mental health, hypocritical and potentially damaging. This echoes reactions from professional bodies, mental health charities and organisations (e.g. BEAT, 2020) who have critiqued the campaign for its use of shaming language. For example, when the campaign was first launched it was described using wording such as ‘war on obesity’ and ‘protect the NHS, save lives’ (Mead, 2020). The latter statement is particularly concerning given that individuals experiencing eating disorders have already been shown to be reluctant to seek help during the pandemic due to worrying that they might be a ‘burden’ to the NHS (Branley-Bell & Talbot, 2020).

Despite aiming to achieve positive changes, the campaign is likely to have been detrimental to some of the population. Clearly, there is a need for future campaigns to avoid negative public responses, minimise harms and maximise the intended benefits. To achieve this, we suggest the following points of action guided by the findings from our research:

- Campaigns must move away from ‘surface-level solutions’ and focus on a more appropriate, complete picture of health and wellbeing. It is vital that campaigns recognise that mental and physical health are intertwined and it is not appropriate to just target physical health without consideration of related mental issues. Failing to encompass mental health components can lead some populations to feel overlooked.
- Campaigns must consider other wider societal issues that may provide a barrier to the behaviour being promoted (e.g. education, financial situation, etc.). A successful campaign will recognise and address these issues wherever possible – this can prevent users reacting negatively to the campaign due to feeling that important issues are being ignored (e.g. cost of healthy diets).
- Campaigns and other public messages must pay careful consideration to the language used, avoiding the use of shaming and/or triggering terminology. Emphasis should also be placed upon overall improved health and not on physical attributes such as weight or body shape. Many negative reactions to the Better Health campaign resulted from perceptions of inappropriate language use.
- Campaigns must involve the public and targetted audiences during design and development. In the instance of the Better Health campaign, it would have been beneficial to identify and involve audiences which may be particularly affected by messages around weight (e.g. individuals with lived experience of eating disorders). This can help to avoid widespread public backlash and potential harm to vulnerable populations. Individuals with lived experience can help to advise on appropriate terminology, highlight wider societal factors and/or behavioural drivers which may have been initially overlooked, and help to provide initial insight into how the campaign may be perceived prior to widespread public release.
- Care must be taken to promote a consistent message across campaigns and sectors. A ‘whole systems approach’ is needed and opportunities for public messages to be perceived as contradictive or hypocritical must be avoided. For example, although some restaurants on the ‘Eat Out to Help Out’ scheme may have offered healthy food choices, the existence of this campaign and the ‘Better Health’ campaign were perceived by many to be contradictory.

Our research has some limitations. Firstly, only Twitter users were included in this study. While Twitter is a valuable resource for accessing public opinions (Branley & Covey, 2017; Highfield, 2016), researchers could also use alternative methods (e.g. surveys, interviews and focus groups) to access more diverse perspectives and ensure that data is captured from users who do not use, or have access to, this platform or technology. Users may also be more likely to tweet when they have a negative opinion, which may have resulted in our sample being more negatively weighted. However, that is not to discount the value of these opinions as a resource from which to learn and develop more effective campaigns in the future.

In conclusion, public reactions to the Better Health campaign were mixed, with some individuals appearing to embrace the campaign and others being more critical due to its perceived inconsistency with other guidance, lack of consideration for mental health and ‘surface-level’ solution to the complex issue of obesity. Our findings show that public health campaigns do have value in promoting positive behaviour change. However, the Better Health campaign in it’s current form could potentially exacerbate mental health difficulties; particularly for those with eating disorders due to it’s focus on weight and perceived ‘fat-shaming’ approach. We have detailed clear points of action to help maximise the positive impacts of future public health campaigns, and minimise unintended harms. In particular, we recommend that the voices of vulnerable groups are centred in the development of future campaigns and a ‘whole systems approach’ is taken to promote better health.
